# Inactivation of CK1α in multiple myeloma empowers drug cytotoxicity by affecting AKT and β-catenin survival signaling pathways

**DOI:** 10.18632/oncotarget.14654

**Published:** 2017-01-14

**Authors:** Sabrina Manni, Marilena Carrino, Martina Manzoni, Ketty Gianesin, Sara Canovas Nunes, Matteo Costacurta, Laura Quotti Tubi, Paolo Macaccaro, Elisa Taiana, Anna Cabrelle, Gregorio Barilà, Annalisa Martines, Renato Zambello, Laura Bonaldi, Livio Trentin, Antonino Neri, Gianpietro Semenzato, Francesco Piazza

**Affiliations:** ^1^ Department of Medicine, Hematology and Clinical Immunology Section, University of Padova, Padova, Italy; ^2^ Venetian Institute of Molecular Medicine, Padova, Italy; ^3^ Department of Oncology and Hemato-Oncology, University of Milano, Milano, Italy; ^4^ Hematology Unit, Fondazione IRCCS Ca' Granda, Ospedale Maggiore Policlinico, Milano, Italy; ^5^ Immunology and Molecular Oncology Unit, Veneto Institute of Oncology, IOV-IRCCS- Padova, Italy

**Keywords:** CK1α, multiple myeloma, lenalidomide

## Abstract

Recent evidence indicates that protein kinase CK1α may support the growth of multiple myeloma (MM) plasma cells. Here, by analyzing a large cohort of MM cases, we found that high CK1α mRNA levels are virtually associated with all MM patients. Moreover, we provided functional evidence that CK1α activity is essential for malignant plasma cell survival even in the protective *niche* generated by co-cultures with bone marrow stromal cells. We demonstrated that CK1α inactivation, while toxic for myeloma cells, is dispensable for the survival of healthy B lymphocytes and stromal cells. Disruption of CK1α function in myeloma cells resulted in decreased Mdm2, increased p53 and p21 and reduced expression of β-catenin and AKT. These effects were mediated partially by p53 and caspase activity. Finally, we discovered that CK1α inactivation enhanced the cytotoxic effect of both bortezomib and lenalidomide. Overall, our study supports a role for CK1α as a potential therapeutic target in MM in combination with proteasome inhibitors and/or immunomodulatory drugs.

## INTRODUCTION

Multiple myeloma (MM), the second most frequent hematologic neoplasia accounting for about 10–15% of all blood tumors [1], is characterized by the progressive growth of malignant clonal plasma cells (PCs) in the bone marrow (BM) where they find a favourable microenvironmental *niche*. Often myeloma cells develop resistance to therapy, likely due to a continuous selection of more biologically aggressive subclones [2].

Among the several signalling pathways regulating MM cell growth, the Wnt/β-catenin and the PI3K/AKT cascades are of particular importance for MM expansion. PCs from MM patients and human MM cell lines (HMCLs) express Frizzled receptors and LRP5/6 co-receptors, as well as high levels of phosphorylated AKT on Ser473, which depends on mTOR activity [3]. Both β-catenin and AKT act as oncogenes and display an increased activity in a wide variety of malignant tumors, including MM [4, 5]. Moreover, BM stromal cells (BMSC) release Wnt or other growth factors which can potentially contribute to MM cell drug resistance [4, 6], triggering β-catenin and the PI3K/AKT signalling [7].

Protein kinase CK1 is a highly conserved family of monomeric serine/threonine kinases composed by seven members (α, β, γ1, γ2, γ3, δ, ε) which display the highest homology in their kinase domain (50–90% identical) with overlapping substrate specificity. CK1 members regulate membrane biology, molecule transport, signal transduction, transcription, translation and DNA-damage response [8–10]. CK1α, which is encoded by the *CSNK1A1* gene, mapping on chromosome 5 at 5q32, regulates the Wnt/β-catenin signalling pathway. CK1α phosphorylates β-catenin at Ser45, priming it for the subsequent protein kinase GSK3-dependent phosphorylation at Ser33/37/Thr41, which tags the protein for proteasome-mediated degradation [11]. However, CK1α may also phosphorylate LRP6, triggering Wnt-mediated intracellular signalling [12].

CK1α is also a regulator of the AKT pathway. It has been reported that in human embryonic kidney cells CK1α phosphorylates DEPTOR (an mTOR inhibitor), which is then targeted to the proteasome, thus activating mTOR-mediated survival pathways [13, 14]. Since mTOR in turn regulates AKT activation [15], CK1α could indirectly modulate AKT function.

CK1α also phosphorylates the tumor suppressor p53 [16] and stimulates the binding of murine double minute chromosome 2 (Mdm2) to p53, therefore inhibiting p53 function [17, 18]. In mouse models, CK1α loss of function in intestinal epithelial cells caused a strong activation of the Wnt pathway, however it did not lead to tumor formation as long as p53 function remained intact [19, 20]. On the opposite, in a murine acute leukemia (AML) model, CK1α loss of function resulted in a dramatic disadvantage for the leukemic clone growth, provided the presence of an intact p53 function [21]. Furthermore, the role of CK1α in mediating tumor cell survival is supported by the finding that treatment with the immunomodulatory drug (iMID) lenalidomide (Lena) induced the E3 ubiquitin ligase CUL4-RBX1-DDB1-CRBN (CRL4^CRBN^)-mediated ubiquitination of CK1α in del(5q) myelodysplastic syndromes (MDS), in which one *CSNK1A1* allele is lost, with degradation of the residual CK1α protein [22].

To inhibit CK1α activity, specific small ATP-competitive molecules have been developed. D4476 (4-[4-(2,3-Dihydro-1,4-benzodioxin-6-yl)-5-(2-pyridinyl)-1H-imidazol-2-yl]benzamide) is a cell-permeant inhibitor specific for CK1. It has been demonstrated that D4476 does not inhibit other important kinases (like ERK2, JNK, MSK1, PDK1 and PKA) and it is the best CK1 inhibitor commercially available [23].

More recently, it has been demonstrated that CK1α also sustains MM cell survival [24]. Here, we investigated *CSNK1A1* mRNA expression in a large microarray dataset of MM cases and analyzed CK1α role in MM cell growth, also in BM microenvironment models. We found that CK1α inhibition/silencing causes cell cycle arrest and apoptosis of MM cells in a p53-Mdm2 dependent manner, overcoming BMSC-dependent protection. Mechanistically, CK1α inhibition caused downregulation of the β-catenin and AKT survival pathways and empowered the cytotoxic and cytostatic effect of bortezomib (BZ) and Lena.

## RESULTS

### CK1α expression and cellular localization is different between MM cells and normal cells

**Figure 1:**
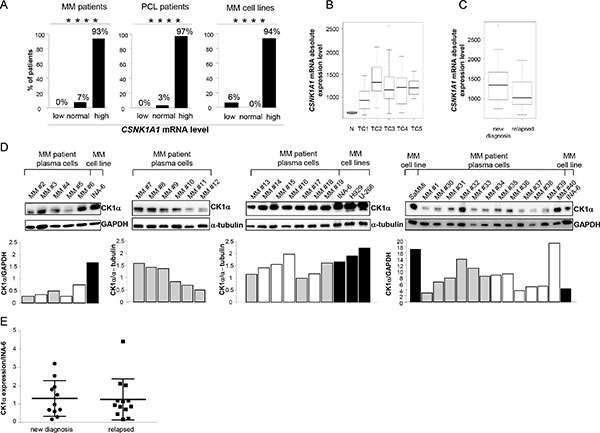
CK1α mRNA and protein expression in MM patients and cell lines (**A**) *CSNK1A1* mRNA expression analysis on 129 MM patients (pts) PCs (left panel), 36 PCL patients PCs (middle panel), 18 MM cell lines (right panel), compared to *CSNK1A1* mRNA expression level of 4 healthy controls PCs. All primary malignant PCs were purified from diagnostic BM aspirates. Samples were stratified according to *CSNK1A1* mRNA expression as follow: normal (value = mean of controls ± 2SD), low (value < mean of controls - 2SD) and high (value > mean of controls + 2SD). The HMCLs used were UTMC2, KMS12BM, LP1, INA-6, CMA-03, DELTA 47, H929, KMS11, OPM2, RPMI-8226, KMS34, MM.1.144, KMS20, KMS26, KMS18, U266, CMA-0306, MM1S, listed according to *CSNK1A1* expression, having UTMC2 the lowest and MM1S the highest expression. Among those, UTMC2 was the only one that had *CSNK1A1* mRNA expression level lower than controls. **** = *p* < 0.0001 according to the Chi square test for trend. (**B**) Box plot of *CSNK1A1* absolute expression level as detected by GEP in 4 healthy donors (N) and different MM TC groups: TC1 (34 pts), TC2 (30 pts), TC3 (40 pts), TC4 (19 pts); TC5 (6 pts). *p* = 1.454 × 10^-6^ was calculated by Kruskal-Wallis test. (**C**) Box plot of *CSNK1A1* absolute expression level as detected by GEP in 17 symptomatic MM and 2 primary PCL patients, included in GSE66293 proprietary dataset [31] investigated at diagnosis and first relapse. Patients were treated with BZ- based therapeutic regimens. No significant difference was observed between at diagnosis and relapsed samples (*p* = 0.212) using Wilcoxon signed-rank test. (**D**) WB analysis (upper panel) and the corresponding densitometry values of CK1α expression in 30 MM patient derived CD138^+^ PCs and in HMCLs INA-6, U-266, H929 and SaMMi. Grey bars represent newly diagnosed patients, white bars represent relapsed patients and black bars are cell lines. GAPDH or α-tubulin was used as a loading control. (**E**) Densitometric analysis of CK1α protein expression of MM patients described in D, grouped in new diagnosis and relapsed MM patients. Values are normalized over CK1α expression of INA-6 cell line.

In most available gene expression profiling (GEP) datasets we found that *CSNK1A1* mRNA is significantly overexpressed throughout the progression from normal to highly malignant PCs (Oncomine™) [25–27]. Also, *CSNK1A1* mRNA was found overexpressed in XBP1s-expressing transformed PCs from transgenic mice [28]. To further validate these data, we investigated GEP data of BM plasma cells obtained from 4 healthy controls, 129 MM, 36 plasma cell leukemia (PCL) patients, and 18 MM cell lines. More than 90% of malignant plasma cells cases overexpressed *CSNK1A1* mRNA compared to controls (Figure 1A). We next performed a correlation between *CSNK1A1* mRNA expression and the different molecular groups included in the TC classification: TC1, characterized by the t(11;14) or t(6;14) with high expression of *CCND1* or *CCND3*; TC2, characterized by absence of IGH translocation, low levels of *CCND1* and hyperdiploid status; TC3, characterized by absence of IGH translocation and *CCND2* expression; TC4, showing high level of *CCND2* and the presence of t(4;14); TC5, expressing the highest level of *CCND2* in association with MAF translocations [29, 30]. *CSNK1A1* mRNA was significantly higher in TC2 samples compared to the other TC groups (Figure 1B). We have also evaluated the absolute transcript levels of *CSNK1A1* in 17 symptomatic MM and 2 primary PCL patients, included in GSE66293 proprietary dataset [31], investigated at diagnosis and first relapse. No significant difference in *CSNK1A1* mRNA expression was observed between these two conditions (Figure 1C). To further corroborate the mRNA data, we next performed a survey of CK1α protein expression in purified CD138^+^ malignant PCs from MM patients and HMCLs showing high CK1α protein levels expression in all the samples analyzed (Figure 1D; Table 1A summarizes the clinical features of the MM patients investigated in this set of experiments). Densitometric analysis of CK1α protein expression confirmed that there is no difference between newly diagnosed and relapsed patients (Figure 1E).

**Table 1:** Clinical and pathological features of MM patients analysed

Sample	Age	Sex	DS Stage	ISS Stage	Paraprotein Type	% PCs	Karyotype	New Diagnoses	Bone Disease
(A)									
MM 1 (SaMMi)	81	F	IA	III	Micromolecular, λ	20	Abnormal karyotype; chr. 1 rearrangement; t(11;14)	Y	N
MM 2	81	F	IA	III	IgG/k	46	Normal	Y	N
MM 3	60	F	IIA	II	IgG/k	80	Normal	N	Y
MM 4	77	F	IIA	I	IgA/k	36.9	59% del(17p)	Y	Y
MM 5	74	M	IA	III	IgG/k	63.8	del(13q)	N	N
MM 6	58	M	IIIA	I	IgG/λ	20	Normal	N	Y
MM 7	72	M	IIA	I	IgG/k	30	del(17p)	Y	Y
MM 8	58	M	IIIA	III	IgA/k	16	Monosomy 13 (90%)	Y	Y
MM 9	71	F	IIIA	III	IgG/k	19	Normal	Y	Y
MM 10	76	M	IIA	III	IgG/k	45	IgH rearrangement	N	N
MM 11	74	M	IIIA	II	IgG/λ	56	Trisomy 1q, 7, 13, 19, 22; tetrasomy 15, del(8p), del(8p), t(2;3)	Y	Y
MM 12	64	M	IIIB	III	IgD/λ	80	Complex karyotype; t(11;14), t(1;14), del(13)	Y	Y
MM 13	71	F	IIA	I	IgG/λ	30	Partial del(1); partial 1q trisomy	Y	Y
MM 14	60	M	IIIA	I	IgG/k	40.6	IgH rearrangement, del(13q)	N	Y
MM 15	68	F	IA	I	IgG/λ	50	Normal	N	N
MM 16	60	F	IA	I	IgA/λ	51.7	Complex karyotype; del(1p); presence of hyperdiploid clones	N	Y
MM 17	67	F	IIIA	III	Non secretory	100	Monosomy 13 (76%)	Y	Y
MM 18	68	F	IIIA	III	Micromolecular, k	80	Normal	N	Y
MM 19	69	M	IIIA	II	Micromolecular, k	60	del(13q), MAF+, IgH-/+	Y	Y
MM 20	71	M	IIIA	III	IgG/λ	90.1	del(Y), del(13q)	N	Y
MM 21	81	M	IIIA	II	Micromolecular, λ	35	NA	Y	Y
MM 22	76	F	IIIA	I	IgG/k	95	Trisomy 5, 6, 9, 11, 15, 21	Y	Y
MM 29	44	M	IIIB	III	IgA/λ	24	Complex karyotype; Monosomy 13, 14, 90% del(17p); Chr. 1 rearrangements, gain of 1q	N	Y
MM30	76	M	IA	NA	IgG/k	60	Normal karyotype, IgH rearr., trisomy 17	Y	N
MM31	72	F	IIIA	III	IgG/λ	100	Complex karyotype, hyperploid; del(13q)	Y	N
MM32	67	M	IIIA	II	IgG/λ	100	NA	Y	Y
MM33	75	M	IIIB	III	IgG/λ	40-100	Hypodiploid , t(14;16), del(13q), gain of 1q	Y	N
MM34	74	M	IIIB	III	Micromolecular, λ	100	IgH-; del(17p), gain of 1q	Y	Y
MM35	56	M	IIIA	NA	IgG/λ	100	NA	N	Y
MM36	42	F	IIIA	III	IgA/k	90	Normal	N	Y
MM37	56	M	IIIA	I	IgA/l	100	Complex karyotype (+1, del(1), dic(1;5), -13,-16, +17. FISH: del(1q), del(13q), del FGFR	N	Y
MM38	70	M	IIA	II	IgG/λ	20	NA	N	Y
MM39	74	M	IIA	II	IgG/k	100	46XY; t(11;14); gain of 1q	N	Y
MM40	75	F	IIIA	II	IgG/k	100	Complex karyotype; del(6p), del(17p), monosomy 10, 12, 13, 15, 16, 17	N	Y
**(B)**									
MM 8	58	M	IIIA	III	IgA/k	16	Monosomy 13 (90%)	Y	Y
MM 14	60	M	IIIA	I	IgG/k	40.6	IgH rearrangement, del(13q)	N	Y
MM 23	59	M	IIIA	I	IgG/k	44	Trisomy 1, 5, 9, 18, 19, 21	Y	Y
MM 24	45	F	IIIA	III	IgG/k	86	90% MAF-/+, Monosomy 13	N	Y
MM 25	54	F	IIA	I	IgG/k	22	t(4;14), Monosomy 13	N	Y
MM 26	74	F	IA	I	IgG/k	26.4	t(14;16)	N	Y
MM 27	76	F	IIA	III	IgG/λ	22.8	Normal	N	Y
MM 28	75	M	IIB	III	IgG/λ	14	36% del(Y), Monosomy 13	Y	Y

MM plasma cells (A) and MM BMSC (B) were isolated from bone marrow of patients listed. Clinical staging was performed according to Durie-Salmon criteria and according to the International Staging System (ISS; albumin levels ≤ or > 35 g/L; β2-microglobulin levels ≤ or > 3.5 mg/L). All features are reported as data at diagnosis. M = Male, F = Female, DS = Durie-Salmon, ISS = International Staging System, PC = Plasma Cell, Y = Yes/Present, N = No/Absent, NA = Not Available.

Previous studies reported that CK1α is detected in all cellular compartments and that its subcellular distribution is cell-cycle dependent [32]. We found that CK1α localized mainly in the cytoplasm of healthy B lymphocytes, while it was present both in the cytoplasm and in the nucleus of malignant PCs from MM and PCL patients and in all the HMCLs tested (Supplementary Figure 1). This different localization of CK1α was present also in a MM cell line, called SaMMi, which was generated in our laboratory from a primary tumor (see Table 2 for details).

**Table 2:** Immunophenotype and cytogenetic features of SaMMi MM cell line

Cell type	CD138	CD38	CD117	CD19	CD56	CD45	Mono κ	Mono λ
**SaMMi**	**+**	**–**	**–**	**–**	**+**	**+**	**-**	**+**
**MM**	**+**	**+/–**	**+/–**	**–**	**+**	**+**	**+/–**	**+/–**

SaMMi was compared with common MM cell lines (MM). Mono κ and Mono λ refer to clonal restriction for κ and λ light chain, respectively. The karyotype of SaMMi is the following:

45,X,-X,idic(1)(p11),der(1)t(1;3)(q25;q21),del(4)(q25),t(4;12)(q26;q13),

der(14)t(11;14)(q13;q32)t(8;14)(q23;q32),der(9)del(9)(p21)t(1;18;9)(q12;p11;q34)[25].ish idic(1)

(p11)(CDKN2C-,CKS1B++),der(1)t(1;3)(CDK2C+,CKS1B+)del(17)(p13.1p13.1)(TP53-,

17p11.1-q11.1+),der(18)t(1;18;9)(CKS1B+)[4].

### CK1α inhibition causes MM cell apoptosis even in the presence of BMSCs

**Figure 2:**
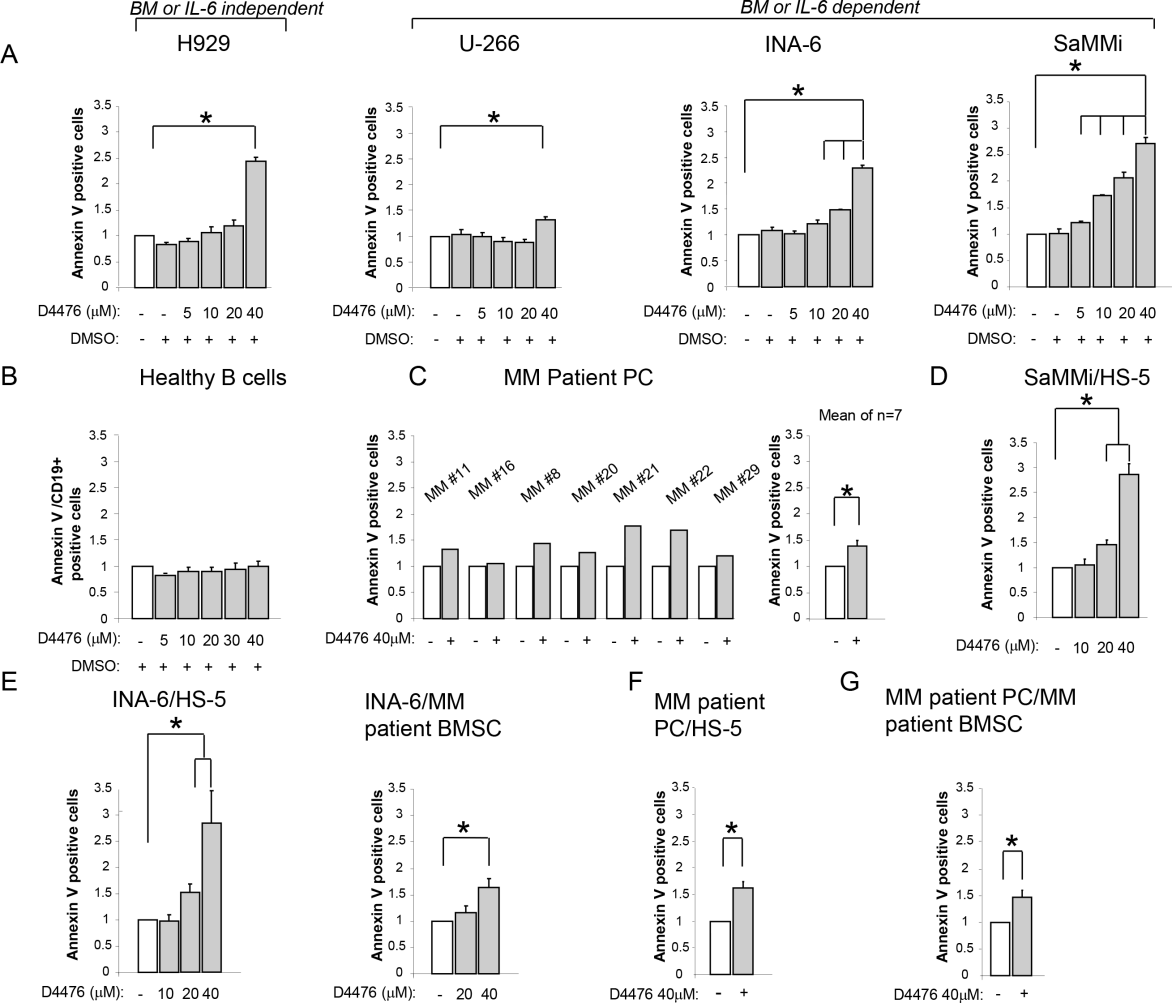
Effects of CK1 inhibition on MM cell survival Quantification of apoptosis through Annexin V staining and FACS analysis in various models of MM cells treated with different concentrations of D4476 (ranging from 5 to 40 μM) for 48 h: (**A**) MM cell lines H929, U-266, INA-6, SaMMi; (**B**) average of 6 purified healthy B cells; (**C**) 7 independent CD138^+^ MM patient PCs (MM#11, MM#16, MM#8, MM#20, MM#21, MM#22, MM#29); (D) SaMMi cultured on HS-5 stromal cells (SaMMi/HS-5); (**E**) INA-6 cultured on HS-5 stromal cells (INA-6/HS-5) and on patient derived BMSCs (INA-6/MM patient BMSC); (**F–G**) patient derived CD138^+^ plasma cells cultured on HS-5 (MM patient PC/HS-5) or on MM patient derived BMSCs (MM patient PC/MM patient BMSC). Data represent the mean ± SEM of 3-6 (A), six (B), seven (C), six (D) four-six (E), two (F) seven (G) independent experiments and are presented as arbitrary values over untreated cells. * indicates *p* < 0.05. DMSO 0.04% was also tested to exclude D4476 vehicle induced toxicity.

To confirm that high levels of CK1α kinase in MM are functionally relevant, we treated MM cells with the CK1 inhibitor D4476 at different concentrations for as long as 48 h. Annexin V/PI staining and FACS analysis showed that MM cells exposed to D4476 underwent a remarkable amount of apoptosis starting from the concentration of 10 μM for INA-6 (the most sensitive MM cell line) reaching 40 μM for H929 and U-266 (the least sensitive). SaMMi cells were also very responsive to D4476-induced apoptosis at a concentration as low as 5 μM. D4476 induced MM cell apoptosis both in IL-6/stroma dependent (INA-6, SaMMi, U-266) and independent (H929) HMCLs. Treatment with the vehicle alone (DMSO) did not cause a significant amount of apoptosis compared to untreated cells (Figure 2A). To test the effects of D4476 in control cells, we treated PBMCs from healthy donors with D4476 at different doses for 48 h and analyzed apoptosis in the CD19^+^ fraction by flow cytometry. The CK1 inhibitor did not cause any significant changes in apoptosis of CD19^+^ healthy B lymphocytes at any concentration used compared to DMSO treated cells, indicating no toxicity for normal B cells (Figure 2B). Also D4476 treated CD138^+^ PCs isolated from seven MM patients underwent, on average, a significant amount of apoptosis compared to untreated cells (Figure 2C). When CK1 inhibition was tested also in a model of protective microenvironment [33] we observed that BMSCs were not able to protect INA-6 or SaMMi MM cells from D4476-induced cell death (Figure 2D–2E). To note, HS-5 stromal cells protected SaMMi from doxorubicin-induced apoptosis (Supplementary Figure 2) showing that also this new cell line is suitable for the BM microenvironment model experiments. CK1 blockade had cytotoxic consequences mainly on malignant PCs, since HS-5 cells were spared by D4476-induced cytotoxicity (data not shown). Most importantly, the apoptotic effect of D4476 was also found in patient-derived PCs co-cultured with HS-5 or patient-derived BMSCs (Figure 2F–2G; see Table 1B for clinical features of patients). To note, even though we observed higher protection by patient-derived BMSCs compared to the HS-5 stromal cell line, D4476 was still able to induce MM cell apoptosis. These results suggest a pro-survival role of CK1 for MM cells also in the BM microenvironment context.

### CK1 blockade associates with activation of a p53-dependent apoptotic cascade

D4476-induced apoptosis was accompanied by increased expression levels of pro-apoptotic proteins (Bax, Bak, cleaved PARP) and decreased expression of the pro-survival molecule Mcl1 (Supplementary Figure 3A). It was previously shown that CK1α promotes p53 degradation in different cell types [16, 17]. Indeed, also in INA-6, U-266, and SaMMi MM cells, D4476 caused an increase of p53 protein levels with a consequent upregulation of its target p21 and a reduction of Mdm2 protein (Supplementary Figure 3B). Altogether, these findings support the view that D4476-induced apoptosis could be mediated by the activation of a p53-dependent response.

### CK1α silencing causes apoptosis and cell cycle arrest

**Figure 3:**
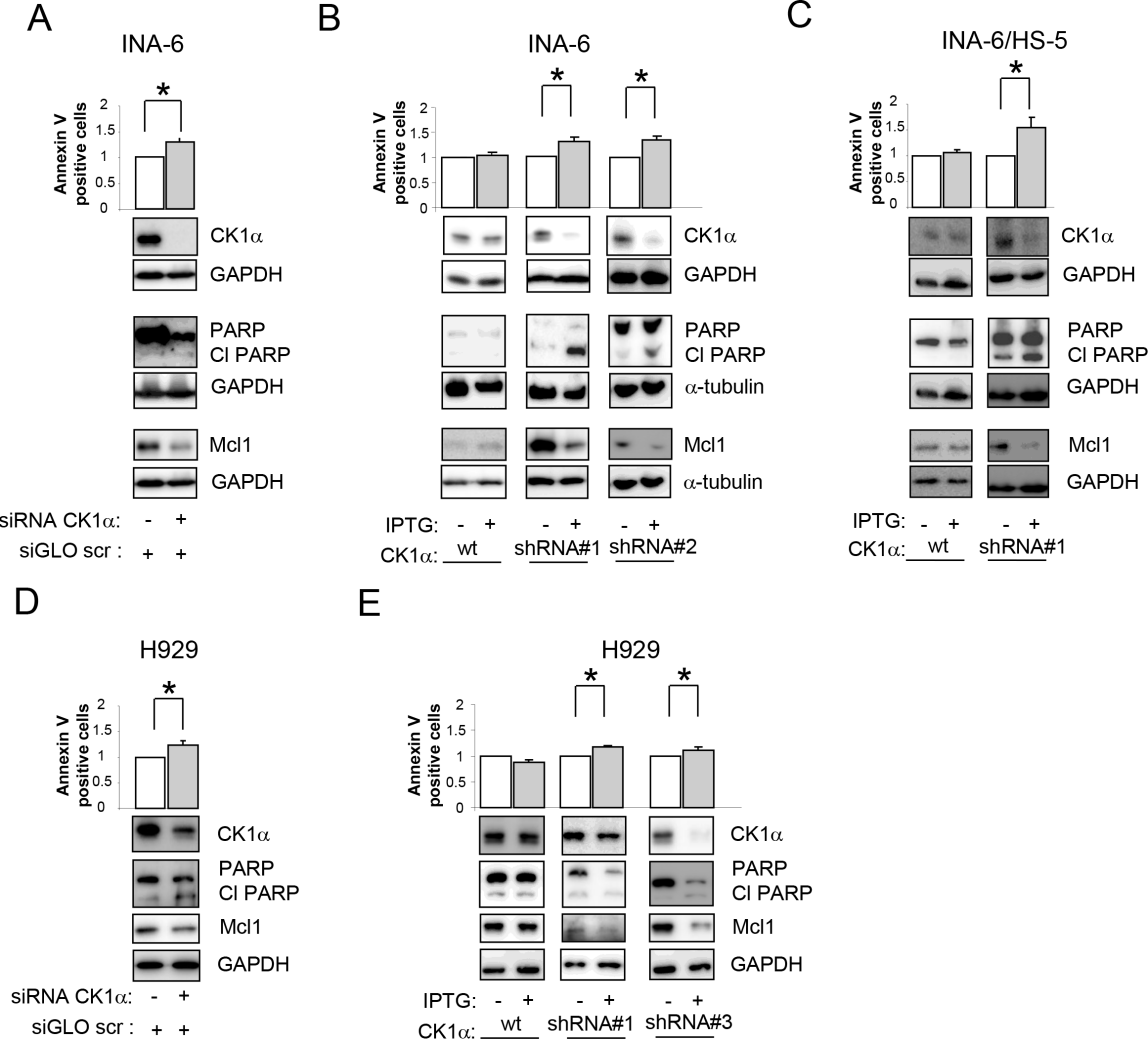
Effects of CK1 inhibition on MM cell survival Quantification of apoptosis through Annexin V/PI staining and FACS analysis (upper panel) and determination of PARP cleavage and Mcl1 protein expression (lower panel) in INA-6 (**A–C**) or H929 (**D, E**) silenced for CK1α. CK1α silencing was obtained with electroporation of ds oligonucleotides directed against CK1α (A, D) or using IPTG inducible CK1α shRNA cellular clones (shRNA#1, shRNA#2, shRNA#3, B,C,E). Cells were treated with IPTG 500 μM (INA-6, and H929 shRNA#1) or 1000 μM (H929 shRNA#3) for 7 days (INA-6 shRNA#1, and H929 shRNA#1 and 2) or 12 days (INA-6 shRNA#2). For the BM microenvironment model (**C**) INA-6 wt and shRNA#1 cells were treated with IPTG 500 μM for 6 days and subsequently plated on HS-5 stromal cells for an additional 4 days in the continuous presence of IPTG. In all experiments, IPTG was added also to wt INA-6 cells and H929 cells as control. GAPDH or α-tubulin was used as loading control. * indicates *p* < 0.05. Data represent the mean ± SEM of three independent experiments and are presented as arbitrary values over untreated cells.

To validate the results obtained with the chemical inhibition of CK1α, we used RNA interference (RNAi) to knockdown CK1α protein in INA-6 and H929 cells. As shown in Figure 3, CK1α knockdown (obtained both with electroporation of double strand (ds) siRNA or through the generation of IPTG inducible *CSNK1A1* specific shRNAs–see methods) determined a boost of apoptosis in INA-6 cells grown alone (Figure 3A–3B), or in the presence of protective stromal HS-5 (Figure 3C). To check the effects of CK1α knockdown also in the BM microenvironment context, we pre-treated INA-6 clones with IPTG for 6 days to induce the silencing of CK1α. Afterwards, control and silenced cells where co-cultured with HS-5 for additional 4 days (in the presence or absence of IPTG). CK1α knockdown determined an increase of apoptosis also in the context of the microenvironment, as shown by the increase in the amount of Annexin V positive cells, a raise of PARP cleavage and decrease of Mcl1 protein expression level (Figure 3C), therefore overcoming stromal cells protection. The apoptotic effect of CK1α silencing was observed also in H929 cells (Figure 3D–3E). In all samples, immunoblot analysis confirmed that CK1α was efficiently knocked down. Of note, IPTG treatment did not change CK1α expression levels and the apoptotic rate in wt INA-6 or H929 cells compared to untreated cells, confirming the efficacy/specificity of the silencing strategy (Figure 3B–3E). The pro-apoptotic effect of CK1α knockdown was further confirmed by cell cycle analysis. CK1α silencing determined an increase in the sub-G1 (apoptotic) phase and a reduction in G0/G1 phase of the cell cycle (Supplementary Figure 4A), accompanied by a reduction of Mdm2 and an increase of p21, (Supplementary Figure 4B) confirming the activation of a p53-dependent cell cycle arrest.

### Inhibition of CK1α leads to a caspase-dependent β-catenin and AKT degradation

**Figure 4:**
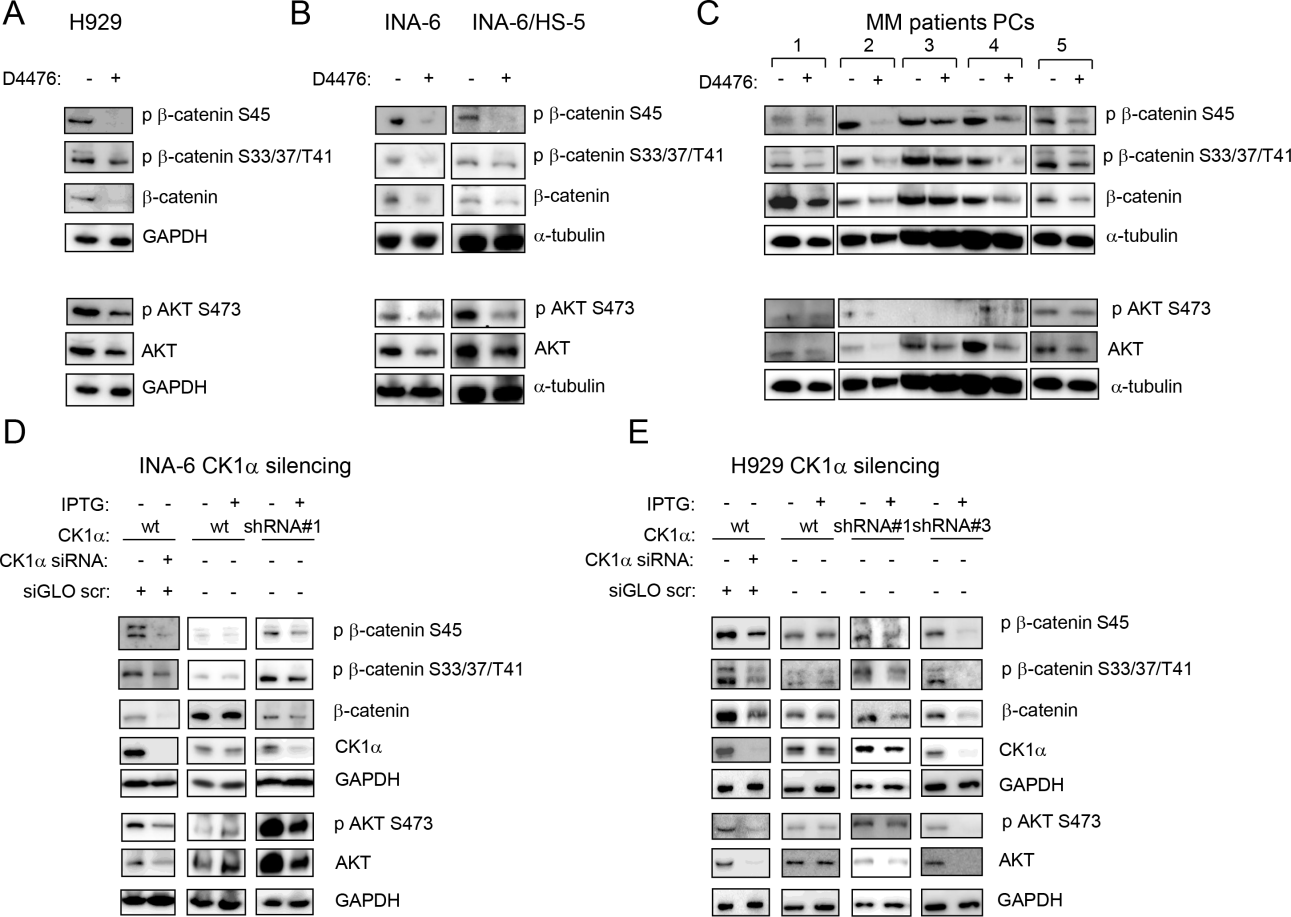
Effects of CK1 inactivation on β-catenin and AKT signalling pathways H929 (**A**), INA-6 grown alone or in co-culture with HS-5 (INA-6/HS-5) (**B**), CD138^+^ patient derived PCs grown alone or on patient-derived BMSCs or HS-5 (**C**) were treated with D4476 40 μM (H929 and patient PCs) or 20 μM (INA-6) for 48 h and the expression of phosphorylated β-catenin on Ser45 (p β-catenin S45), phospho β-catenin on Ser33/37/Thr41 (p β-catenin S33/37/T41), phospho AKT on Ser473 (p AKT S473), total β-catenin and AKT was evaluated by WB. GAPDH or α-tubulin was used as loading control. In C: 1 = MM#11, 2=co-culture of MM#12 on BMSC obtained from MM#28; 3 = co-culture of MM#12 on BMSC obtained from MM#8; 4 = co-culture of MM#12 on HS-5; 5 = co-culture of MM#20 on BMSC obtained from MM#28. The same analysis was performed in INA-6 (**D**, left panel) or H929 (**E**, left panel) cells electroporated with ds oligonucleotides against CK1α, in INA-6 wt or INA-6 CK1α shRNA#1 cellular clone treated with IPTG 500 μM for 1 week (D, middle and right panels) or in H929 wt, H929 CK1α shRNA#1 and shRNA#3 clones, treated with IPTG 500 μM (H929 wt and shRNA#1) or 1000 μM (shRNA#3) for 1 week (E, right panel).

**Figure 5:**
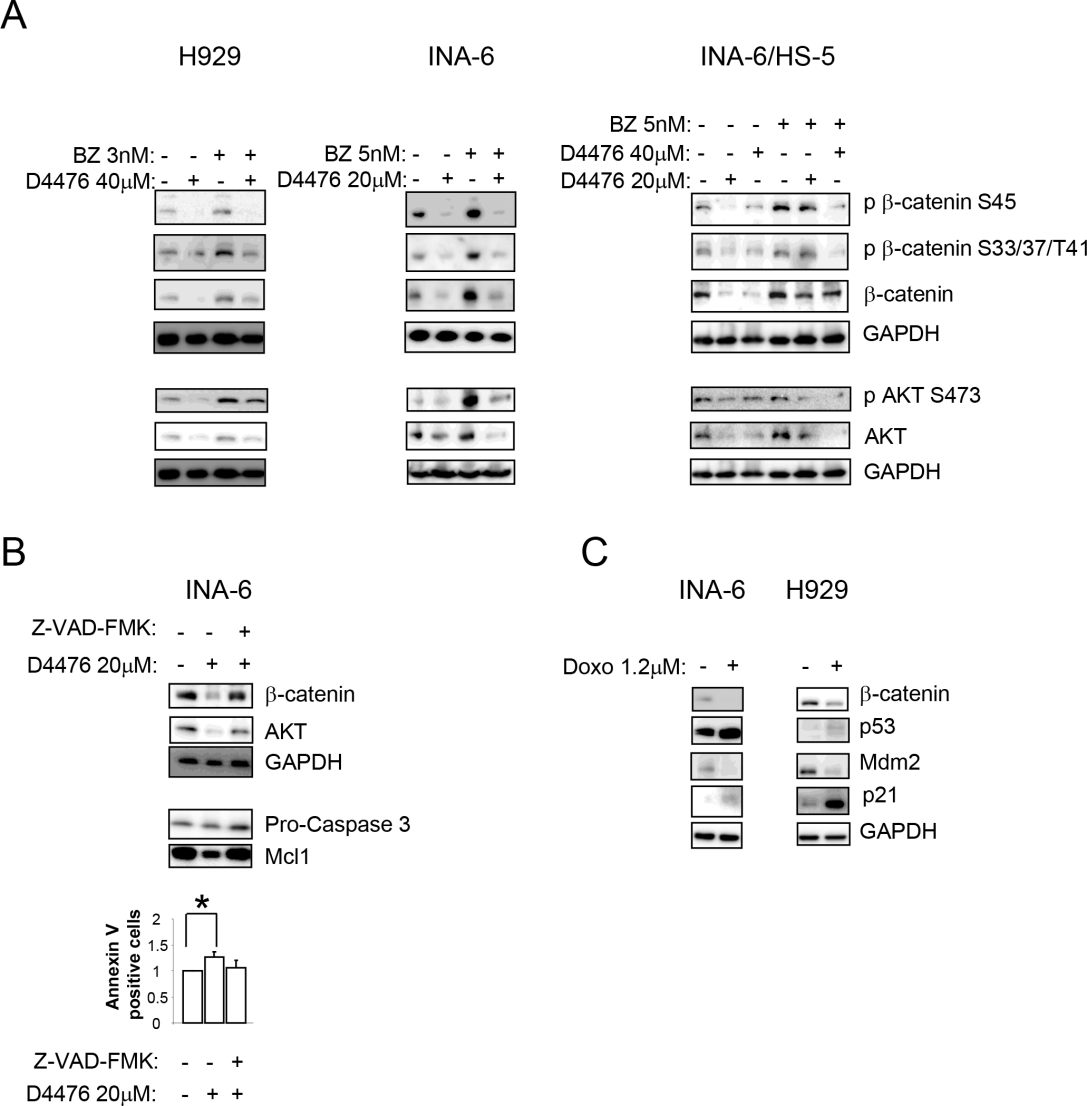
The D4476-dependent reduction of β-catenin and AKT is independent from the proteasome and is mediated by caspase and p53 (**A**) Expression of phospho β-catenin on Ser45 (p β-catenin S45), phospho β-catenin on Ser33/37/Thr41 (p β-catenin S33/37/T41), phosphorylated AKT on Ser473 (p AKT S473) and total β-catenin and AKT in H929 (left panel) and INA-6 cells, grown alone (middle panel) or in co-culture with HS-5 (INA-6/HS-5, right panel), treated with D4476 for 48 h and BZ for 18 h or the combination of the two compounds at the concentrations indicated in the Figure. (**B**) Expression of total β-catenin, AKT, Pro-caspase 3 and Mcl1 proteins in INA-6 cells treated with D4476 20 μM in association with Z-VAD-FMK for 48 h. In the combination treatment, cells were pretreated with Z-VAD-FMK 2 μM for 1 h before the addition of D4476. (**C**) INA-6 and H929 cells were treated with doxorubicin (Doxo) 1.2 μM for 18 h. β-catenin, p53, Mdm2, and p21 were evaluated by WB. In all experiments GAPDH was used as loading control.

Since β-catenin and AKT signalling pathways are crucial for MM pathobiology, and given the role of CK1α in modulating their activity in other cell types, we sought to investigate the role of CK1α on β-catenin and AKT transduction cascades in MM. H929, INA-6 and MM patient-derived plasma cells (grown alone or in co-culture with HS-5 or MM BMSCs) were treated with the indicated concentrations of D4476 and WB analysis was carried on. After CK1 inhibition, a reduction of phosphorylated β-catenin on Ser45 and on Ser33/37/Thr41 and of phosphorylated AKT on Ser473 was observed in all the tested cells (Figure 4A–4C). However, unexpectedly, a reduction of total β-catenin and AKT protein levels was also observed, which could account for the lowering of the phosphorylated forms. RNAi of CK1α in two MM cell lines led to similar effects (Figure 4D–4E). To test if the observed reduction of β-catenin and AKT induced by CK1α inactivation was proteasome dependent, we treated H929 and INA-6 cells grown alone or in co-culture with HS-5 with D4476 for 48 h and with the proteasome inhibitor BZ for 18 h. D4476 prevented BZ-induced accumulation of β-catenin and AKT, indicating that the observed D4476-induced β-catenin and AKT decrease was not mediated by the proteasome (Figure 5A).

Another AKT or β-catenin degradation mechanism could involve caspases and the tumor suppressor p53 [34, 35]. Previous studies in other cell types, showed a reduction of β-catenin upon increasing expression and activity of p53 [35]. Moreover, other works described a caspase-dependent (mainly caspase 3, 6 and 8) and apoptosis-induced cleavage of β-catenin [36, 37]. Therefore, to test if this mechanism could be responsible of β-catenin and AKT degradation upon CK1α inhibition also in MM cells, we treated INA-6 cells with D4476 and the pan-caspase inhibitor Z-VAD-FMK. The addition of Z-VAD-FMK, prevented the D4476-induced reduction of β-catenin and AKT. The efficacy of the caspase inhibitor was confirmed by Annexin V/PI staining, Pro-caspase 3 and Mcl1 protein detection by WB (Figure 5B). Next, we checked if other p53-inducing agents could correlate with a reduction in β-catenin expression in MM. Remarkably, doxorubicin determined an upregulation of the p53 axis (p53 and p21 increase, reduction of Mdm2 protein level) with concomitant decrease in total β-catenin level in INA-6 and H929 (Figure 5C). Taken together, these results suggest that p53 and caspase-mediated apoptosis could be responsible for the D4476-induced reduction of β-catenin and AKT in MM cells.

### CK1 inhibition boosts bortezomib induced cytotoxicity

Since CK1α promotes MM cell survival, we sought to investigate if its inhibition could empower the cytotoxicity exerted by bortezomib. To this aim, we first assessed the extent of apoptosis of MM cells exposed in culture to D4476 and BZ. D4476 caused an increase of BZ-induced apoptosis in the BZ sensitive U-266, H929 and INA-6 MM cells (Supplementary Figure 5A–5C). To evaluate the role of CK1α in BZ resistance, we also treated RPMI-8226 (a less BZ sensitive cell line) with subtoxic doses of BZ (5 nM and 7.5 nM) in association with D4476. In this cell line, higher doses of BZ (10 nM) are needed to obtained BZ-induced cytotoxicity (Supplementary Figure 5D). CK1 inhibition in association with BZ was able to cause a moderately higher level of apoptosis compared to the single treatments and overcome BZ resistance in these cells. Furthermore, we have treated the UTMC2 cell line (which is reported to be BZ resistant [38]), with D4476. CK1 inactivation caused a strong dose dependent cytotoxicity in UTMC2 cells without an additive role with BZ. Moreover D4476 cooperated with BZ in inducing cell death also in the model of BM microenvironment of INA-6 grown in co-cultures with HS-5 or patient-derived BMSCs (Supplementary Figure 5F–5G). Importantly, CK1 inhibition was able to overcome patient BMSCs-induced resistance to BZ (Supplementary Figure 5G). To determine if the inhibition of CK1 and the proteasome could cause either an additive or a synergic effect in terms of cell growth arrest, we performed MTT assay evaluating cell viability after treating H929 cells with increasing concentration of BZ and D4476 and with the combination of the two compounds. The results showed that treatment of MM cells with BZ and the CK1 inhibitor was strongly synergic (Supplementary Figure 5H), as judged by the calculated Combination Index (CI) well below 1 (0.560). Moreover, we evaluated CK1α expression in MM patients with regards to BZ sensitivity, by analyzing *CSNK1A1* mRNA expression in the public dataset GSE9782 [39]. Patients included in this study were treated with BZ and the dataset consisted of 169 patients evaluable for response. A slight significant difference was evidenced between 85 responder and 84 not responder MM patients, showing higher expression levels of *CSNK1A1* in not responder patients (Supplementary Figure 5I).

### CK1α is degraded by lenalidomide and its inhibition/silencing reinforces lenalidomide cytotoxicity

**Figure 6:**
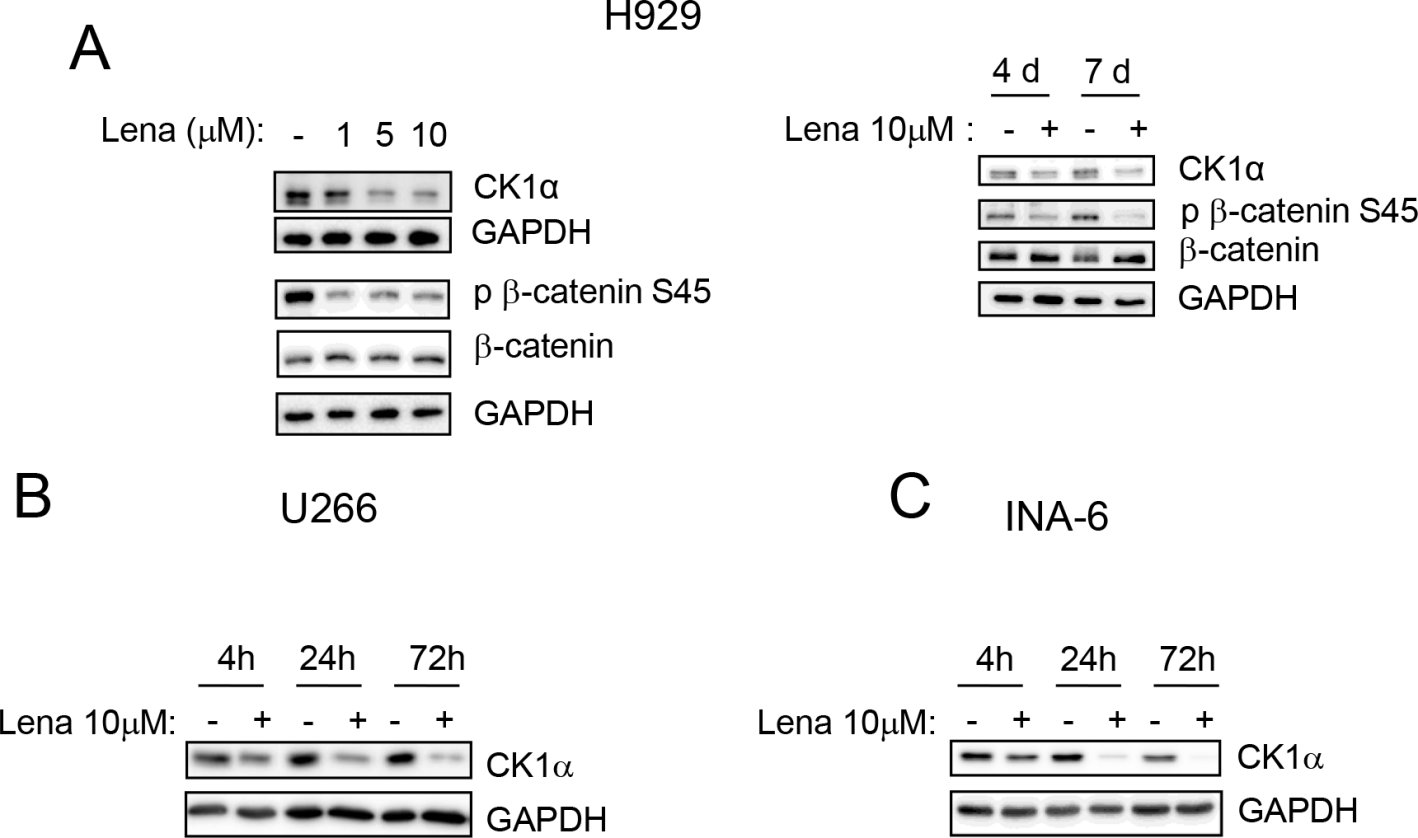
Lenalidomide determines CK1α protein reduction in a dose and time dependent manner (**A**) H929 cells were treated with Lena at different concentrations (1, 5, 10 μM) for 7 days (left panel) or with Lena 10 μM for 4 (4d) and 7 days (7d). U-266 (**B**) and INA-6 (**C**) were treated with Lena 10 μM for 4 h, 24 h and 72 h. CK1α, phosphorylated β-catenin on Ser45 (p β-catenin S45) and total β-catenin were evaluated by WB. GAPDH was used as loading control.

To investigate if CK1α inhibition could modify iMIDs induced cytotoxicity, we first treated H929 MM cells with different concentrations of Lena for 4 and 7 days and we confirmed a pro-apoptotic and an antiproliferative/cytostatic effect of Lena in MM cells (Supplementary Figure 6). We next established that Lena causes a dose- and time-dependent degradation of CK1α protein in H929 with a subsequent reduction of its kinase activity (a decrease phosphorylation of its target phosphorylated Ser45 β-catenin was shown) (Figure 6A). A time-dependent Lena-induced degradation of CK1α was confirmed also in U-266 and INA-6 cell lines at even earlier time points (4 h treatment) (Figure 6B, 6C).

**Figure 7:**
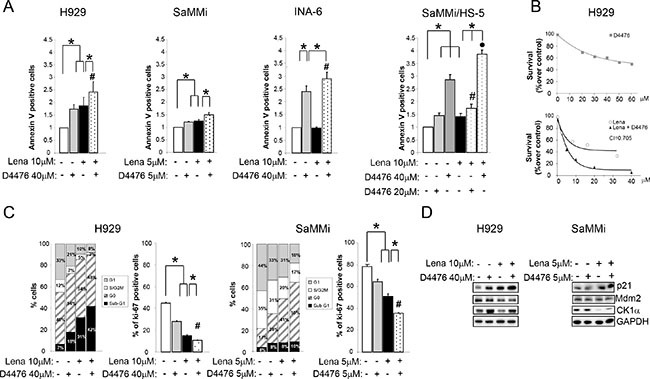
Lenalidomide empowers D4476-induced apoptosis and cell cycle arrest MM cells were treated with Lena for 3 days (SaMMi, INA-6) or 7 days (H929), with D4476 for the last 48 h and with the combination of the two compounds at the doses indicated in the Figure. (**A**) Annexin V/PI staining and FACS analysis of the Lena sensitive cell lines, H929, SaMMi grown alone, or in co-culture with HS-5 (SaMMi/HS-5) and of the Lena resistant INA-6. (**B**) Synergistic effect of D4476 and Lena in reducing cell viability. Upper graph: dose response curve of H929 incubated for 72 hours with increasing concentrations of D4476 (grey squared curve). Lower graph: dose-response curve of cells incubated for 1 week with increasing concentrations of Lena alone (white round curve) or Lena plus D4476 (black triangle curve). In the combination treatment, D4476 was added for the last 72 h. Cell viability was assessed with MTT test and reported as percentage over DMSO treated cells. IC50 for D4476 was 60 μM and for Lena 10 μM. The CI between D4476 and Lena was calculated as to be 0.705. (**C**) Cell cycle analysis with Ki-67/PI staining (left panel) and total Ki-67 (right panel) in H929 and SaMMi cells. * indicates *p* < 0.05; ^#^ indicates *p* < 0.05 between samples treated with D4476 together with Lena and cells treated only with D4476. Data represent the mean ± SEM of three independent experiments. (**D**) Expression analysis of p21, Mdm2 and CK1α in H929 and SaMMi. GAPDH was used as loading control.

Next, Lena sensitive (H929, SaMMi) and insensitive (INA-6) cells were treated with Lena 10 μM for 3 or 7 days. Forty-eight hours before harvesting, D4476 was added to the cell culture. D4476 strikingly cooperated with Lena in inducing MM cell death causing an increase of apoptotic cells compared to single treatments (Figure 7A). Interestingly, CK1α inhibition overcome Lena resistance in INA-6 cells. The therapeutic potential of Lena and D4476 was demonstrated also in models of myeloma BM microenvironment. Indeed, Lena potentiated the cytotoxic effect of D4476, overcoming BM protection also in SaMMi cells grown on HS-5 (Figure 7A, right panel).

To test if the two drugs acts in a synergistic mode, we performed the MTT viability assay calculating the CI. H929 cells were treated with increasing doses of Lena or D4476 and with the combination of the two compounds. The CI was 0.705 suggesting a synergy between the CK1 inhibitor and Lena in reducing cell viability (Figure 7B).

Next we evaluated the effect of D4476 and Lena on cell cycle. D4476 plus Lena caused a decrease in the percentage of cells in G1 and in S/G2M phases and an increase in the frequency of sub-G1 apoptotic cells, compared to the single treatments (Figure 7C). Also Ki-67 staining showed a significant reduction of active proliferating cells in the combined treatment (Figure 7C). Moreover, a raise of p21 and a decline of Mdm2 protein levels were stronger in the combination treatment compared to the single treatments (Figure 7D).

**Figure 8:**
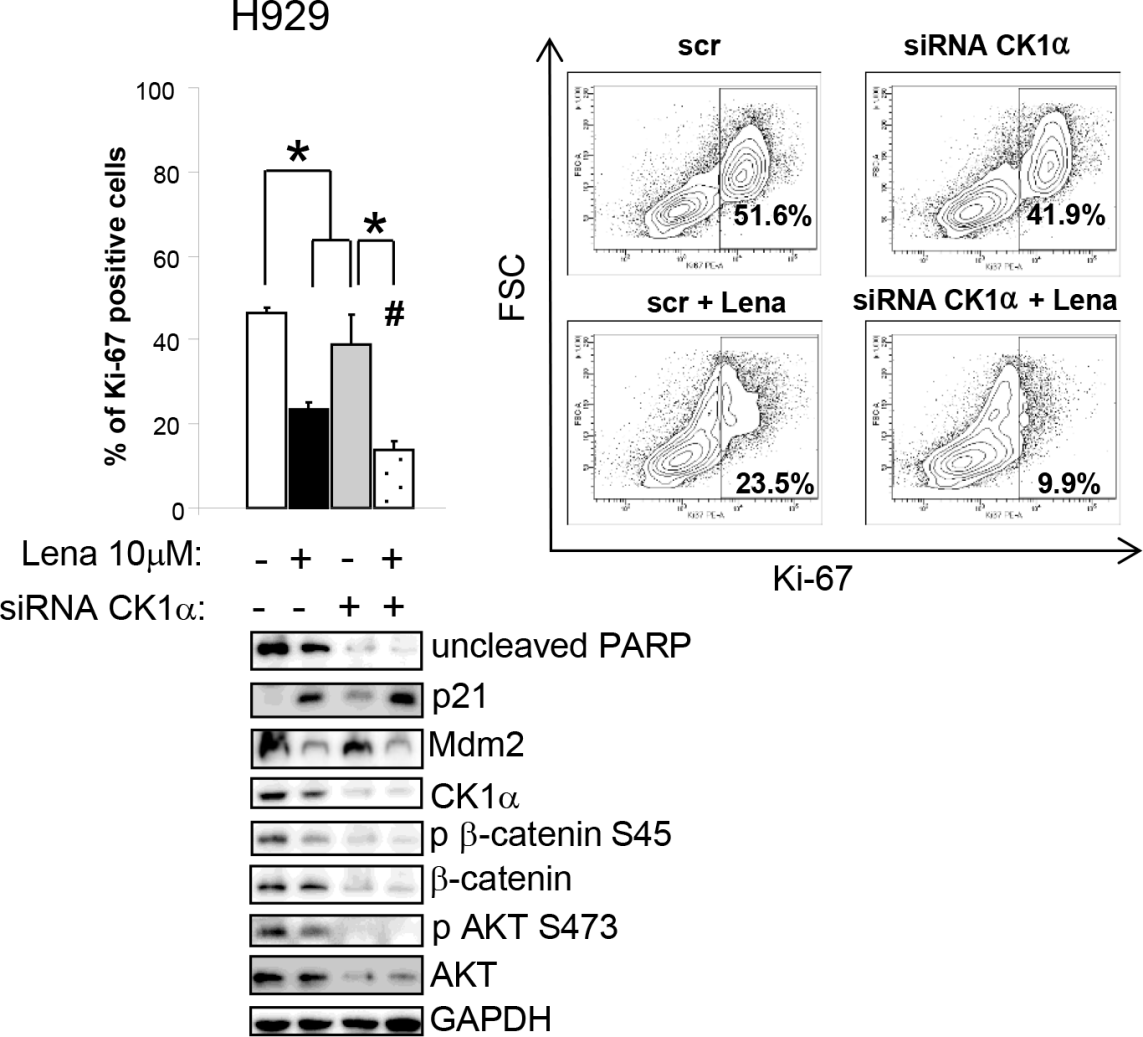
Lenalidomide cooperates with CK1α silencing in inducing apoptosis and cell cycle arrest, modulating β-catenin and AKT survival signalling Ki-67 positive H929 cells after transfection with CK1α siRNAs and siGLO green indicator and treated for 3 days with Lena (average of five independent experiments). * indicates *p* < 0.05. ^#^ indicates *p* < 0.05 between samples treated with Lena alone or in combination of CK1α silencing. Data represent the mean ± SEM of five independent experiments. The right panel shows a representative Contour Plot. Bottom panel: expression of phosphorylated β-catenin on Ser45 (p β-catenin S45), phosphorylated AKT on Ser473 (p AKT S473), total β-catenin and AKT. GAPDH was used as loading control.

Next, we treated CK1α-silenced (with siRNA against *CSNK1A1)* and control H929 cells with Lena 10 μM for 3 days. The combination of Lena and CK1α silencing determined a stronger reduction of active proliferating cells as judged by a marked decline in the percentage of Ki-67 positive cells as compared to single treatments. An increase in apoptosis and the derangement in the cell cycle were also confirmed by a reduction in the uncleaved PARP, Mdm2 protein and a subsequent increase in p21. CK1α silencing empowered Lena induced CK1α protein degradation with subsequent further reduction in its activity on Ser45 β-catenin. Moreover, the combination of CK1α silencing and Lena also effectively decrease AKT phosphorylation and protein level (Figure 8).

## DISCUSSION

In the present study we investigated the expression of CK1α in MM and provided evidence of its role in sustaining MM cell survival and proliferation. We also demonstrated that CK1α controls the levels of β-catenin and AKT, likely preventing their p53/caspase-mediated degradation cascade. We confirmed that CK1α is a target of Lena in MM cells and showed that its inactivation cooperates with BZ and Lena in inducing MM cell death, even in the protective *niche* of the BM microenvironment.

In a recent work, Hu *et al*. demonstrated that CK1α sustains MM cell survival [24]. However, no studies have investigated whether CK1α inactivation, especially in the MM BM microenvironment, could affect the cytotoxic effects of novel drugs, such as Lena, in the therapy of this hematological malignancy.

According to previous studies [24], we confirmed, in a larger and representative panel, that *CSNK1A1* mRNA is overexpressed in MM patients. Interestingly, we demonstrated for the first time a differential *CSNK1A1* mRNA expression between the TC groups. In particular, higher levels of *CSNK1A1* were associated with TC2 patients, who are characterized by the hyperdiploid status that involves several odd chromosomes including chromosome 5 where *CSNK1A1* is located. Among the 30 TC2 cases investigated, 26 (87%) showed extra copies of chromosome 5 (data not shown), thus suggesting that a gene dosage effect could be partially responsible of the high expression of the *CSNK1A1* gene (Figure 1B).

To the best of our knowledge, this is the first report analyzing CK1α mRNA and protein expression in a large cohort of MM patients both at diagnosis and relapse. To note, we did not observe substantial differences between these two patient groups, suggesting that CK1α levels could not correlate with the clonal evolution of MM plasma cells. We also demonstrated an atypical nuclear diffuse/microspeckled pattern localization in MM cells, pointing to a myeloma-specific function likely in regulating gene transcription and/or chromatin DNA methabolism [13, 32].

The pro-apoptotic effects and alteration of cell cycle related to CK1α inactivation in MM cells support its pro-survival function in this malignancy. Both CK1α inhibition and silencing were able to overcome the BM microenvironment-dependent protection (Figures 2 and 3). By contrast, CK1α inhibition did not influence HS-5 stromal cell or healthy B lymphocytes mortality, suggesting that this effect could be restricted to malignant MM cells.

Unexpectedly, we found that β-catenin levels decreased upon CK1α blockade. This was not prevented by proteasome inhibition, but it was rescued by caspase blockade. Similarly, CK1α inhibition or silencing was accompanied by AKT kinase decrease, in line with what expected according to the CK1α-mediated inhibition of the AKT inhibitor DEPTOR [14]. Similarly to what demonstrated in lymphoma cells [34], we provided evidence that, also in myeloma cells undergoing p53-dependent apoptosis, AKT is downregulated by a caspase-dependent mechanism.

Moreover, it is known that both AKT [40, 41] and CK1α [18] can phosphorylate and activate Mdm2 with the consequent degradation of p53. In addition, AKT decreases caspase 9 activity by phosphorylation on Ser196 [42], thus promoting cell survival. A reduction in AKT protein level could therefore downmodulate Mdm2 phosphorylation with the subsequent p53 and caspase activation, in a positive feedback loop. The final effect would be the arrest of cell cycle and activation of apoptosis. Since MM cells rely on both β-catenin and AKT, their downregulation achieved with CK1α inactivation could be therapeutically relevant. To this regard, the reduction of BZ-induced AKT and β-catenin accumulation (a possible drawback of BZ) achieved upon CK1α inhibition may potentially overcome BZ resistance (Figure 5). Indeed, CK1α inhibition synergically potentiated BZ toxicity in MM (Supplementary Figure 5). To note *CSNK1A1* mRNA expression is higher in BZ resistant patients, suggesting the possibility that this kinase might influence the sensitivity to BZ. Importantly, CK1α inactivation reduced β-catenin and AKT proteins also in the presence of Lena, and effectively cooperates with this drug in inducing apoptosis and growth arrest, also in the BM microenvironment.

Taken together, our data support an oncogenic role of CK1α in MM, likely by impinging on critical microenvironment dependent survival pathways (Wnt/β-catenin and PI3K/AKT). Therefore, it would be worth pursuing to consider the design of novel selective CK1α inhibitors to be used in the clinical setting in association with conventional or novel anti-myeloma agents.

## MATERIALS AND METHODS

### Patients and cell cultures

PBMC, MM and HS-5 cell lines, primary BMSCs from patients were isolated and cultured as previously described [31, 33, 43]. Malignant CD138^+^ PCs and healthy B cells were isolated with EasySep™ kits (STEMCELL Technologies, USA), after achieving informed consent according to the declaration of Helsinki. The internal Institutional Board approved the use of human material. SaMMi cell line was obtained from a 81 year old woman with a 20% PCs in the bone marrow. CD138^+^ SaMMi PCs were cultured in DMEM 20% FBS with the addition of IL-6 2.5 ng/ml. Co-cultures of HS-5 or BMSCs with INA-6 or patient derived CD138^+^ PCs were stabilized as described in [33]. The clinical features of the patients analyzed and the immunophenotype/karyotype of SaMMi are detailed in Tables 1 and 2.

### GEP and data analysis

The study was performed in a cohort of 165 patients, representative of all the major forms of plasma cell dyscrasia. This dataset, publicly available at the NCBI Gene Expression Omnibus repository (accession #GSE66293), includes 4 normal controls (Voden, Medical Instruments, Italy), 129 MM, 24 primary PCL (pPCL), 12 secondary PCL (sPCL) patients and 18 HMCLs [31]. With the exception of sPCL, the cohort consists of newly diagnosed patients. PCs were purified from BM samples using CD138 immunomagnetic microbeads (MidiMACS system, Miltenyi Biotec, CA, USA). Samples were characterized for the presence of the most frequent chromosomal translocations and the ploidy status based on fluorescence *in situ* hybridization (FISH) evaluation criteria [44]: 48 showed an HD status; 34 were characterized by the t(11;14) or t(6;14) translocations; 19 had the t(4;14) translocation; 6 had either the t(14;16) or t(14;20) translocations; and 22 did not fall into any of the other groups. Details regarding the source of all HMCLs have been previously reported [31, 45, 46] and recently authenticated by STR profiling and/or gene mutational analyses. For gene expression analysis, samples were profiled on the GeneChip Human Gene 1.0 ST array (Affymetrix, CA, USA) as described [47]. The raw intensity expression values were processed by Robust Multi-array Average procedure (RMA) [48] with the re-annotated Chip Definition Files from BrainArray libraries version 19.0.0, available at http://brainarray.mbni.med.umich.edu.

### Cytokines and chemicals

IL-6 and IPTG were from Sigma-Aldrich (Italy); BZ and Lena from Selleck chemicals (USA); D4476 (4-[4-(2,3-Dihydro-1,4-benzodioxin-6-yl)-5-(2-pyridinyl)-1*H*-imidazol-2-yl]benzamide) from abcam (UK); Z-VAD-FMK from Enzo Life Science (UK).

### Evaluation of growth and apoptosis

Apoptosis was assessed by Annexin V/Propidium Iodide (PI) staining (IMMUNOSTEP, Spain) [43]. FACS analysis was performed using a FACSCanto™Cell Cytometer and the FACSDiva™ software (Becton-Dickinson, Italy).

### Assessment of drug concentration-effect and calculation of the combination index (CI)

H929 cells were plated in triplicate into 96 well plates in 100 μl media. D4476, BZ and lenalidomide was added for 72 h (D4476 and BZ) or 1 week (Lena) alone or in combination. Cell viability was analyzed with 3-(4,5-dimethylthiazol-2-yl)-2,5-diphenyltetrazolium bromide (MTT). Cells were washed by centrifugation, resuspended in 100 μl of growing media containing 0.25 mg/ml MTT and left in the incubator for 2–3 h. Formazan blue precipitates were dissolved in DMSO and absorbance was read at 560 nm. CI was calculated as described in [43].

### Cell cycle analysis

5 × 10^5^ cells were fixed in ice cold ethanol 70% v/v in PBS for 1 h. After washing in PBS (2 times), samples were stained with PI (50 μg/ml) and RNaseA 0.2 mg/ml (Sigma-Aldrich, Italy) for 30 min. Ki-67/PI staining was performed using FITC- or PE-conjugated Ki-67 (BD Pharmingen, Italy). FACS analysis was performed as above.

### Immunofluorescence

It was performed as in [43] with the following antibodies: anti-CK1α (abcam, UK), secondary Alexa-Fluor 594-conjugated goat anti-rabbit (Life technologies, Italy). Specimens were mounted in Vectashield medium with DAPI (Vector Laboratories, USA) and analyzed using Leica TCS SP5 confocal microscope, oil objective 63x with the LAS Advances Fluorescence Leica Application Suite software (Italy).

### Western blot (WB)

WB was performed as described [49]. Antibodies used were the following: CK1α, PARP, Mcl1, Ser45 β-catenin, Ser33/37/Thr41 β-catenin, total β-catenin, Ser473 AKT, total AKT (Cell signaling Technology, MA, USA); Mdm2 (Millipore, Italy), GAPDH (Ambion, USA); α-tubulin (Sigma-Aldrich, Italy); Bak (Merck, MA, USA); Bax, p21, p53 (Becton Dickinson, Italy); Caspase 3 (Enzo Life Science, UK). Images were acquired using the Image Quant LAS 500 chemiluminescence detection system (GE Healthcare, USA). Densitometric analysis was performed with Quantity One software (Bio-Rad, Italy).

### mRNA silencing

RNAi was performed with nucleofection of ds siRNA and by the generation of inducible shRNA cell lines.

### Nucleofection

Electroporation of INA-6 and H929 was performed as described in [50] with siGLO Green scrambled siRNAs and CK1α targeting siRNAs ON-TARGETplus SMARTpool siRNA (Thermo Scientific, USA). CK1α target sequences were: GCGAUGUACUUAAACUAUU; GGAAUCAUUAGGAUAUGUU; AGAGUAACAUGA AAGGUUU; GGCUAAAGGCUGCAACAAA. Cells were harvested at 72 h after nucleofection.

### shRNA lentiviral transduction

INA-6 and H929 were transduced with the IPTG inducible lentiviral particles carrying *CSNK1A1*-specific shRNA (pLKO_IPTG_3XLacO, Sigma-Aldrich, Italy). Three independent shRNAs sequences were chosen (TRCN0000006044, named shRNA#1, TRCN0000006287, named shRNA#2, TRCN0000006042, named shRNA#3). 2 × 10^4^ cells were infected with a multiplicity of infection of 5, using the spinfection method (centrifuged at 1000 g for 45 min at 32°C) in the presence of 8 μg/ml polybrene (Sigma-Aldrich, Italy). Puromycin selection (0.5 μg/ml) was initiated two days after transduction. Once a cellular clone was established, to induce CK1α silencing, cells were incubated with 500/1000 μM IPTG every two/three days for a total of one (in the case of shRNA#1 and shRNA#3) or two (in the case of shRNA#2) weeks, time lapse in which we obtained the best results in terms of CK1α knockdown efficacy.

### Statistical analysis

Data obtained were evaluated for their statistical significance with the two-tail paired Student's *t* test or ANOVA analysis of variance with *post-hoc* corrections, with the Chi square test for trend. Conventional statistical procedures were applied using standard packages of the R software. Values were considered statistically significant at *p* values below 0.05.

## SUPPLEMENTARY MATERIALS FIGURES


