# Supplementary material for: Inactivation of CK1α in multiple myeloma empowers drug cytotoxicity by affecting AKT and β-catenin survival signaling pathways
Source: Oncotarget. 2017 Jan 14;8(9):14604–19. doi: 10.18632/oncotarget.14654 (PMC5362429; doi:10.18632/oncotarget.14654)
Supplement: Supplementary file 1 [file oncotarget-08-14604-s001.pdf]

Inactivation of CK1α in multiple myeloma empowers drug cytotoxicity by affecting AKT and β-catenin survival signaling pathways

Supplementary Materials

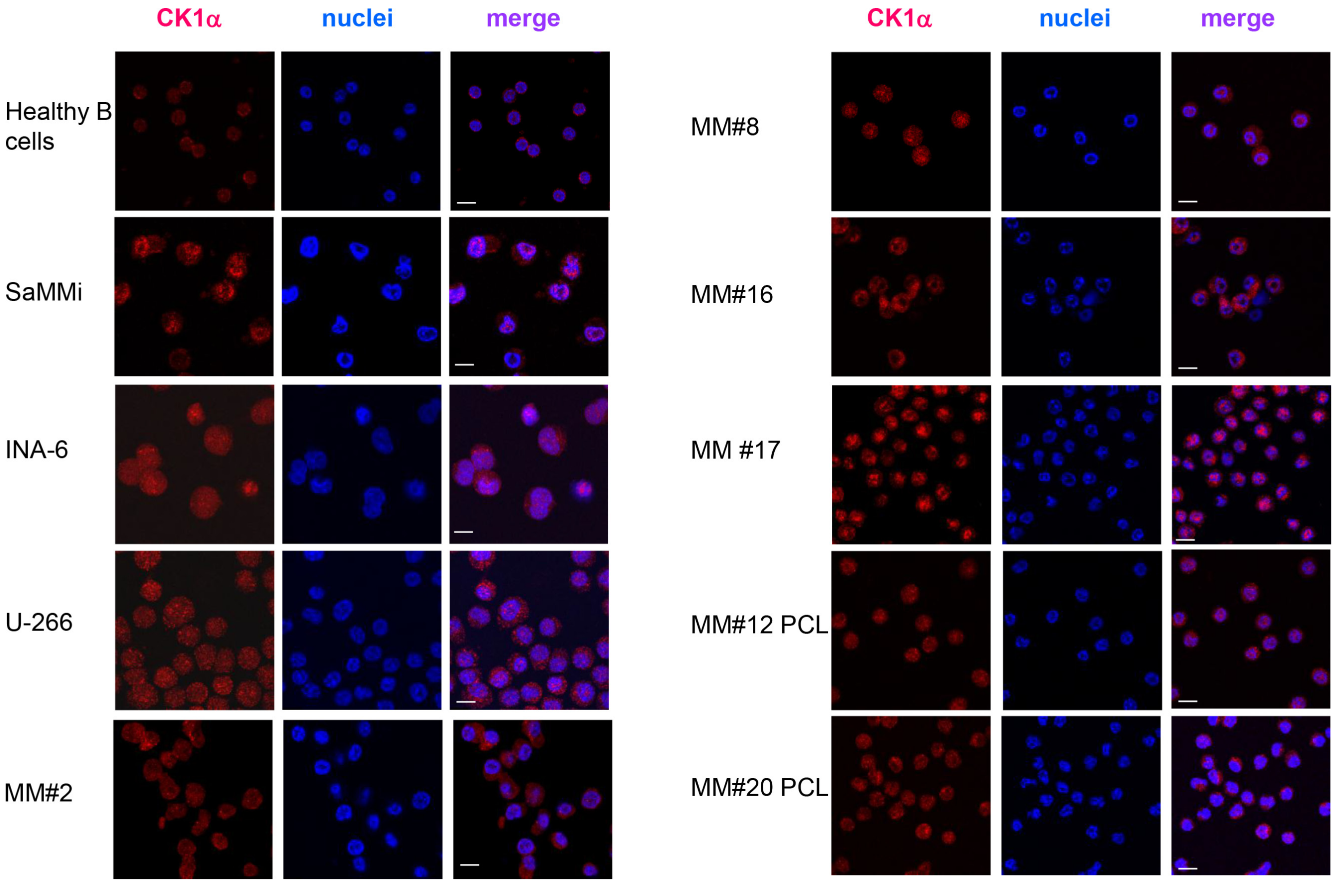

Supplementary Figure 1: CK1α expression and localization in MM cells and controls. Immunofluorescence staining of CK1α cellular localization (red) in purified B healthy lymphocytes (Healthy B cells), in SaMMi, INA-6 and U-266 cell lines, in 4 patient derived CD138+ PCs (MM#2, MM#8, MM#16, MM#17) and in CD138+ purified PCs from two PCL cases (MM#12, MM#20). Nuclei are stained with DAPI (blue). For all the images 63x oil objective was used. Scale bar: 10 µm.

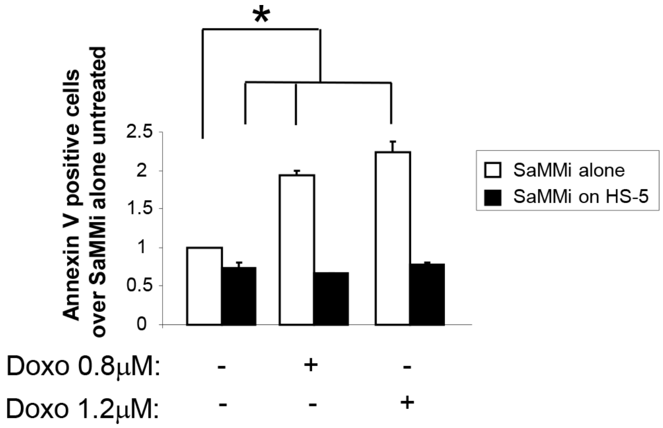

Supplementary Figure 2: HS-5 stromal cells protects SaMMi MM cells from doxorubicin induced cytotoxicity. Quantification of apoptosis through Annexin V/PI staining and FACS analysis of MM cells SaMMi grown alone (white bars), or in co-cultures with HS-5 (black bars), treated with doxorubicin (Doxo) 0.8 μM and 1.2 μM for 18 h. Data represent the mean ± SEM of 3 independent experiments. * indicates $p < 0.05$.

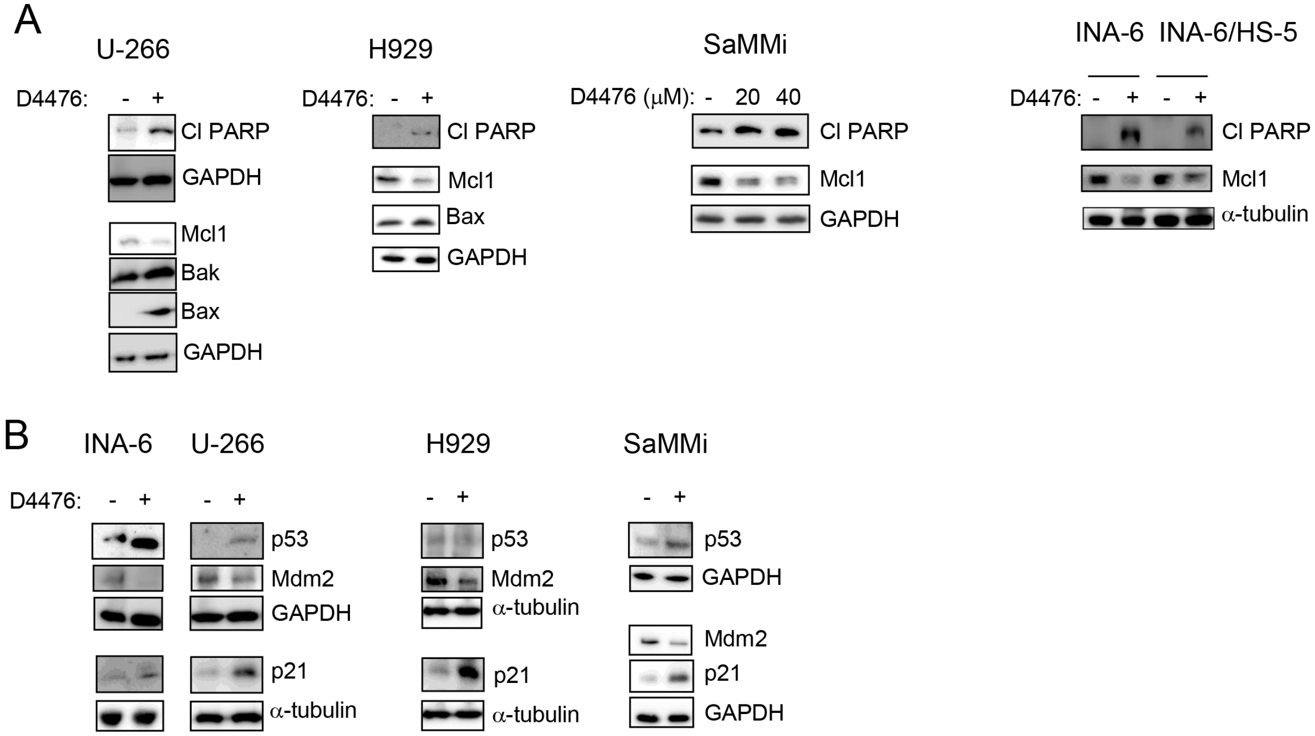

Supplementary Figure 3: CK1α inhibition caused MM cell death in a p53 dependent manner. (**A**) WB analysis of PARP cleavage and Mcl1, Bak and Bax expression in U-266, H929, SaMMi and INA-6 cells grown alone or in co-culture with HS-5 (INA-6/HS-5) treated with D4476 20 μM (INA-6 cells) and 40 μM (U-266, H929 and SaMMi cells). (**B**) WB analysis of p53, Mdm2 and p21 protein expression in INA-6, U-266, H929 and SaMMi cells treated with D4476 20 μM (INA-6) and 40 μM (U-266, H929 and SaMMi). GAPDH or α-tubulin was used as loading controls.

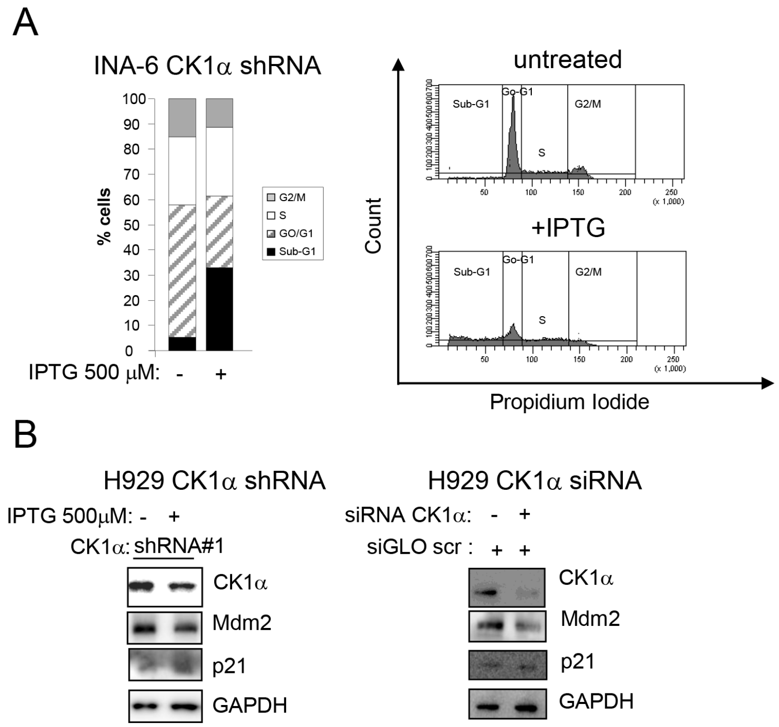

Supplementary Figure 4: CK1α silencing caused apoptosis and cell cycle arrest. (**A**) Quantification of cell cycle phases with PI staining and FACS analysis in INA-6 CK1α shRNA clone#2 treated with IPTG 500 μM for two weeks. Left panel: bar graph representing the average of three independent experiments of the sub-G1 (black), G0/G1 (striped gray), S (white) and G2/M (gray) phases of the cell cycle. Right panel: representative FACS histogram plot. (**B**) Western blot analysis of H929 shRNA clone#1 treated with IPTG 500 μM for 1 week (left panel) or H929 cells silenced with *CSNK1A1* directed ds oligonucleotides. The cell cycle dependent protein p21 and Mdm2 are analyzed by WB. GAPDH was used as loading control.

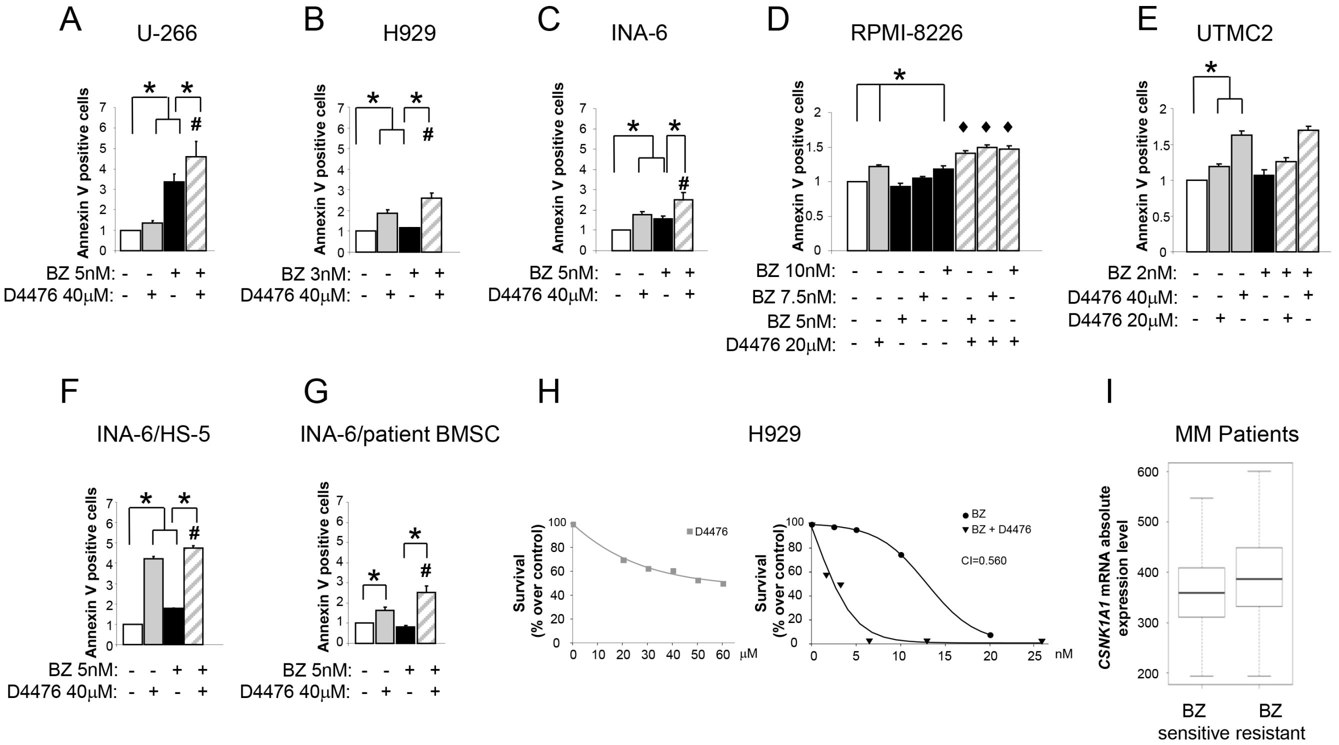

Supplementary Figure 5: Effects of CK1α and proteasome inhibition on MM cell survival. Evaluation of apoptosis through Annexin V/PI staining and FACS analysis of U-266 (**A**), H929 (**B**), INA-6 (**C**), RPMI-8226 (**D**), UTMC2 (**E**), INA-6 co-cultured with HS-5 (INA-6/HS-5) (**F**) or with patient-derived BMSC (INA-6/patient BMSC) (**G**) treated with D4476 (grey bars) for 48h and BZ for 18 h (black bars) and the combination of the two compounds (grey striped bars) at the concentrations indicated in the Figure. Histogram bars represent mean ± SEM of three-six experiments and are presented as arbitrary values over untreated cells. * indicates $p < 0.05$. # indicates $p < 0.05$ between samples treated with D4476 alone and BZ together with D4476. ◊ indicates $p < 0.05$ between samples treated with the combination of D4476 and BZ, compared with D4476 alone or with the corresponding dose of BZ. (**H**) Synergistic effect of D4476 and BZ in reducing cell viability. Left graph: dose response curve of H929 incubated for 72 hours with increasing concentrations of D4476 (grey squared curve). Right graphs: dose-response curve of cells incubated for 72 h with increasing concentrations of BZ alone (black round curve) or BZ plus D4476 (black triangle curve). Cell viability was assessed with MTT test and reported as percentage over DMSO treated cells. IC50 for D4476 was 60 µM and for BZ 12.89 nM. The CI between D4476 and BZ was calculated as to be 0.560. (**I**) Box plot of *CSNK1A1* absolute expression level as detected by GEP in 169 patients treated with BZ and evaluable for response, included in the public dataset GSE9782 [1]. Samples were subject to replicate gene expression profiling using the Affymetrix 133A/B microarray. A median expression value was calculated on the 8 probe sets identifying *CSNK1A1*. A slight significant difference ($p = 0.0199$) was evidenced between 85 responder to BZ and 84 not responder MM patients, showing higher expression levels in not responders.

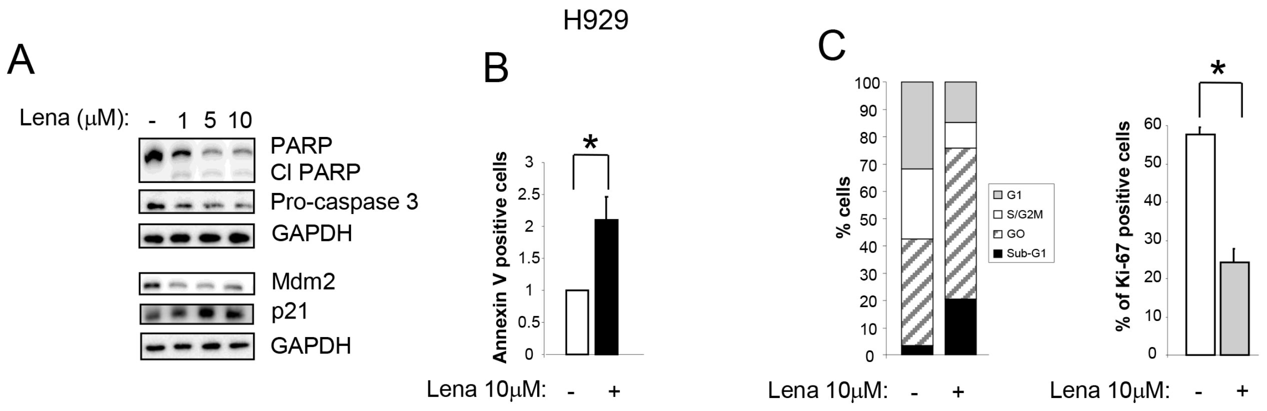

Supplementary Figure 6: Lenalidomide caused cytotoxic and cytostatic effects on MM cells. H929 cells were treated with Lena at different concentration (1, 5, 10 μM) for seven days. (**A**) Increase of PARP cleavage, reduction of Pro-caspase 3 and Mdm2, raise of p21 protein expression were evaluated by WB. GAPDH was used as loading control. Annexin V (**B**) and Ki-67/PI (**C**) staining of H929 cells treated with Lena 10 μM for 7 days. The histogram on the left panel shows the average of three independent experiments of the sub-G1, G0, S/G2M and G1 phases of the cell cycle, while the bar graph on the right side shows the mean ± SEM of total Ki-67 positive cells in the absence or presence of Lena. The graphs showed increased Annexin V/PI positive cell, enhanced sub-G1 apoptotic cell population, reduction of G1, S/G2M and prolonged G0 population after Lena treatment.

REFERENCES

1. Mulligan G, Mitsiades C, Bryant B, Zhan F, Chng WJ, Roels S, Koenig E, Fergus A, Huang Y, Richardson P, Trepicchio WL, Broyl A, Sonneveld P, et al. Gene expression profiling and correlation with outcome in clinical trials of the proteasome inhibitor bortezomib. Blood. 2007; 109:3177–3188.
